# Lateral Femoral Cutaneous Nerve Block or Wound Infiltration Combined with Pericapsular Nerve Group (PENG) Block for Postoperative Analgesia following Total Hip Arthroplasty through Posterior Approach: A Randomized Controlled Trial

**DOI:** 10.3390/jcm13092674

**Published:** 2024-05-02

**Authors:** Giuseppe Pascarella, Fabio Costa, Alessandro Strumia, Alessandro Ruggiero, Luigi Maria Remore, Tullio Lanteri, Anton Hazboun, Ferdinando Longo, Francesca Gargano, Lorenzo Schiavoni, Alessia Mattei, Felice Eugenio Agrò, Massimiliano Carassiti, Rita Cataldo

**Affiliations:** 1 Unit of Anesthesia and Intensive Care, Fondazione Policlinico Universitario Campus Bio-Medico, 00128 Rome, Italy; g.pascarella@policlinicocampus.it (G.P.); f.costa@policlinicocampus.it (F.C.); l.remore@policlinicocampus.it (L.M.R.); t.lanteri@policlinicocampus.it (T.L.); a.hazboun@policlinicocampus.it (A.H.); f.longo@policlinicocampus.it (F.L.); f.gargano@policlinicocampus.it (F.G.); l.schiavoni@policlinicocampus.it (L.S.); a.mattei@policlinicocampus.it (A.M.); f.agro@policlinicocampus.it (F.E.A.); m.carassiti@policlinicocampus.it (M.C.); r.cataldo@policlinicocampus.it (R.C.); 2 Unit of Anesthesia and Intensive Care, Department of Medicine, Università Campus Bio-Medico, 00128 Rome, Italy; alessandro.ruggiero@unicampus.it

**Keywords:** total hip arthroplasty, analgesia, anesthesia, regional anesthesia, nerve block, postoperative pain

## Abstract

**Background:** Pericapsular nerve group (PENG) block, although effective for pain management following total hip arthroplasty (THA), does not cover skin analgesia. In this randomized controlled trial, we compared the effectiveness of PENG block combined with lateral femoral cutaneous nerve (LFCN) block or wound infiltration (WI) on postoperative analgesia and functional outcomes. **Methods:** Fifty patients undergoing posterior-approached THA under spinal anesthesia were randomly allocated to receive LFCN block with 10 mL of 0.5% ropivacaine or WI with 20 mL of 0.5% ropivacaine. In both groups, PENG block was performed by injecting 20 mL of 0.5% ropivacaine. Primary outcomes were static and dynamic pain scores (0–10 numeric rating scale) measured in the first 24 h after surgery. Secondary outcomes included postoperative opioid consumption, functional assessment and length of hospital stay. **Results:** Postoperative static NRS of patients receiving LFCN was higher than that of patients receiving WI at 6 h but lower at 24 h, with a median (IQR) of 3 (2–4) vs. 2 (1–2) (*p* < 0.001) and 2 (2–3) vs. 3 (3–4) (*p* = 0.02), respectively. Static pain scores at 12 h did not show significant differences, with an NRS of 3 (2–4) for WI vs. 3 (3–4) for LFCN (*p* = 0.94). Dynamic pain and range of movement followed a similar trend. No significant differences were detected in other outcomes. **Conclusions:** LFCN block was not inferior to WI for postoperative analgesia and functional recovery in association with PENG block during the first postoperative day, although it had worse short-term pain scores. Based on these results, it is reasonable to consider LFCN block as a valid alternative to WI or even a complementary technique added to WI to enhance skin analgesia during the first 24 h after THA. Future studies are expected to confirm this hypothesis and find the best combination between PENG block and other techniques to enhance analgesia after THA.

## 1. Introduction

Pericapsular nerve group (PENG) block is a fascial block first described in 2018 by Girón Arango and colleagues to treat hip fracture-related pain [1]. It targets periarticular sensory branches derived from the femoral, obturator and accessory obturator nerves innervating the anterior hip capsule [2]. Since 2021, many trials have been published demonstrating its efficacy in managing perioperative pain after hip arthroplasty without affecting functional recovery [3]. However, PENG block does not involve other anatomical structures affected by postsurgical stress, such as skin, periarticular fascias, muscles and posterior capsula. In particular, among all of these structures, skin has been demonstrated to have a higher concentration of nociceptors involved in hip replacement-related pain [4].

Lateral femoral cutaneous nerve (LFCN) block has been shown to contribute to cutaneous anesthesia of hip surgery incisions, although not completely [5,6]. However, these were the results of studies performed on healthy volunteers and do not take count of tissues response to surgical stress, including local inflammatory reactions which could involve tissues near the wound, although not included in the surgical incision line. Not by chance, the positive impact of LFCN block on postoperative analgesia after postero-lateral-approached THA has been confirmed by clinical investigations [7,8].

Although some authors suggest the use of LFCN combined with PENG to enhance postoperative analgesia after THA [9,10], the impact of this association has not yet been sufficiently investigated. At the same time, wound infiltration (WI) has been demonstrated to be effective in managing postoperative analgesia in different kinds of surgeries, including hip surgery [11,12,13].

For these reasons, we conducted a randomized controlled trial to compare the efficacy of PENG block combined with LFCN block or WI, which is our current standard treatment to cover skin analgesia following posterior THA.

## 2. Methods

### 2.1. Enrollment

**Figure 1:**
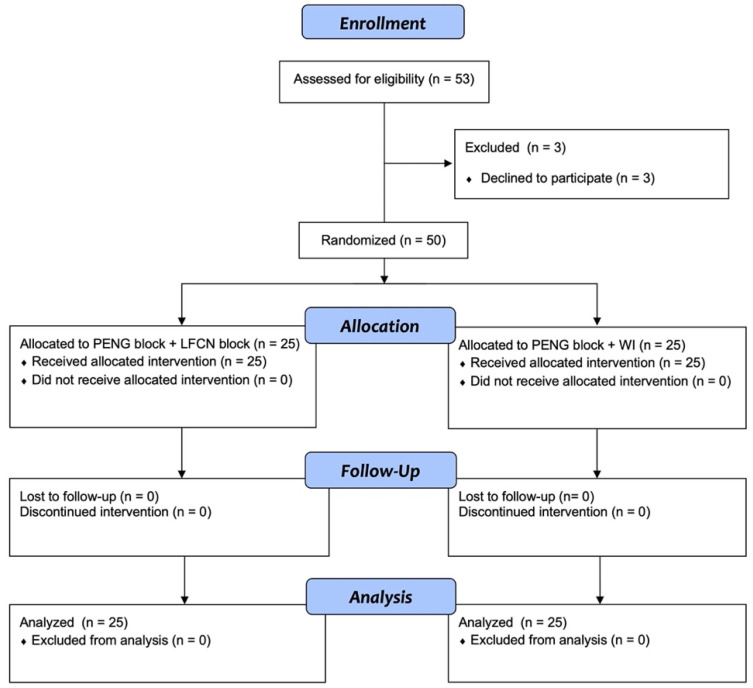
CONSORT flow diagram. CONSORT indicates Consolidated Standards of Reporting Trials.

This study was approved by the Ethics Committee of Campus Bio-Medico University Hospital in Rome (protocol number 34.22, date of approval: 24 May 2022) and registered on ClinicalTrials.gov (NCT05432011, Principal Investigator: Giuseppe Pascarella, Date of registration: 20 June 2022) before the first patient was enrolled. Enrollment was performed from 1 July 2022 to 18 May 2023, offered preoperatively to adults undergoing primary hip arthroplasty at the Day Surgery Department of Fondazione Policlinico Universitario Campus Bio-Medico, Italy, aged ≥ 18 y and ASA physical status 1–3. Patients with allergies to local anesthetics, infection of the puncture site, lack of signing of informed consent, weight < 30 kg, age < 18 years old, ASA physical status IV, dementia or cognitive impairment were excluded from this study. Fifty eligible patients were randomly allocated into two groups to receive a PENG block combined with an LFCN block or WI (Figure 1).

Randomization was achieved using computer-generated lists in blocks of eight with a 1:1 ratio, and treatment allocation was concealed using consecutively numbered, sealed, opaque envelopes. All patients underwent total hip replacement performed in the morning by the same surgical team using a posterior approach. Every patient was informed of the sequence of procedures during anesthesia and surgery, and written informed consent was obtained before enrollment.

Moreover, as part of a preoperative checklist, we always asked patients to describe both the anesthetic and surgical procedure they are undergoing in order to verify their comprehension.

In both groups, patients received mild sedation with 0.03 mg.kg^−1^ intravenous midazolam. Before surgery, 1 g acetaminophen, 30 mg ketorolac and 8 mg dexamethasone were given intravenously (i.v.) as multimodal pre-emptive analgesia. Spinal anesthesia was chosen as the main anesthetic technique. It was performed by injecting 16 mg of ropivacaine 0.5% through a 27G Whitacre needle at the L2–L3 or L3–L4 interspace with the patient in a sitting position and with the help of preprocedural ultrasound spinal evaluation [14,15].

### 2.2. Interventions

Patients were scheduled to receive WI or LFCN at the end of surgery.

**Figure 2:**
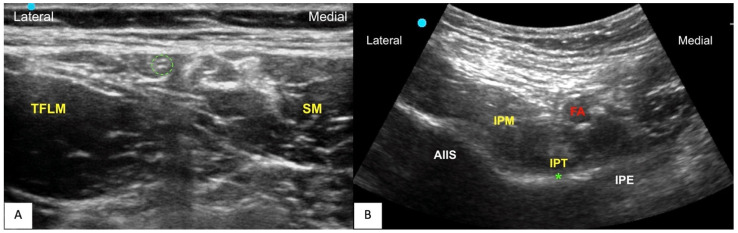
**Regional Anesthesia Techniques:** (**A**) **Lateral femoral cutaneous nerve (LFCN) block.** Green dashed line: LFCN; TFLM: tensor fasciae latae muscle; SM: sartorius muscle (**B**) **PENG Block:** IPT: iliopsoas tendon; IPE: iliopubic eminence; asterisk (green *): injection target; FA: femoral artery; IPM: iliopsoas muscle; AIIS: anterior inferior iliac spine.

WI was performed by the surgeon, injecting 20 mL of 0.5% of ropivacaine in the subcutaneous tissue. Ultrasound-guided LFCN block was performed in the post-anesthesia recovery room (PACU). A linear probe was used to identify the LFCN lying between the sartorius muscle and tensor fasciae latae muscle, and then the needle (50 mm Stimuplex Ultra 360, BBraun, Melsungen, Germany) was inserted from lateral to medial to target the nerve. After a negative aspiration test, 10 mL of 0.5% ropivacaine was injected perineurally (Figure 2A). LFCN block success was assessed using an ice test on the lateral aspect of thigh at dismission from PACU.

Moreover, all patients received PENG block in the PACU block according to the technique originally described by Girón-Arango [1]. A curvilinear probe was used. The needle (80 mm Stimuplex Ultra 360, BBraun, Melsungen, Germany) was inserted lateromedially until it reached the space between the iliopsoas tendon and periosteum, situated on the lateral side of the iliopubic eminence (IPE) (Figure 2B). Following confirmation via a negative aspiration test, 20 mL of 0.5% ropivacaine was injected in the plane beneath the iliopsoas muscle (IPM) to obtain transverse spread under the muscular plane with the tendon lifted [16].

PENG block and LFCN block were always performed by the same four anesthetists, (GP, FC, TL, AH) experts in regional anesthesia.

### 2.3. Postoperative Management

Both groups received the same postoperative multimodal analgesia, which included acetaminophen 1 g i.v. every 6 h and ketorolac 60 mg i.v. every 24 h, as recommended by international guidelines [17]. In addition, patient-controlled analgesia was provided i.v. (morphine bolus = 1 mg; lockout interval = 8 min).

### 2.4. Outcomes Assessment

At 6, 12 and 24 postoperative hours, patients were asked to indicate perceived static (at rest) and dynamic (hip adduction) pain using a 0–10 NRS (0 no pain, 10 worst imaginable pain).

Functional recovery of the hip joint was evaluated through the postoperative range of movement (ROM) together with the ability to perform physiotherapy and ambulation. The ROM was analyzed through active hip flexion, within the range of 0° to 90°, measured with a protractor, at 6, 12, and 24 h postoperatively.

For each time point, quadriceps strength was evaluated by asking the patient to extend the leg to exclude motor paralysis due to PENG block. Furthermore, the ability to start physiotherapy and ambulate thanks to the help of a walker during the first postoperative day was recorded. In case of inability, it was specified if it was related to motor block or pain. The length of hospital stay was also recorded. We recorded any complications or side effects, including local infection, vascular puncture, nausea and/or vomiting, dizziness and respiratory depression. Outcome assessment was performed by the same group of clinicians (AS, LR, AR), blinded to patients’ group allocation.

### 2.5. Statistical Analysis

To calculate the sample size, we focused on the primary hypothesis that postoperative analgesia after PENG block combined with LFCN is not inferior to PENG block with WI. Despite the absence of similar clinical trials at the time of our protocol study, we estimated the density of pain scores (mean 2; SD 1) based on the database of our previously published work regarding the use of PENG block combined with WI for THA [18].

To simulate power, we employed the truncated Gaussian distribution spanning from 0 to 10, with a standard deviation of 1, and a mean of 2 for the PENG + WI group. Based on these parameters and assuming a two-sided significance level of 5%, we conducted 10,000 simulations, each with a sample size of 25 per group. With a total sample size of 50 subjects, we possess 90% power to identify group disparities in pain as minimal as approximately 1. Statistical analysis and visual presentation were obtained using GraphPad Prism 8 software (GraphPad Software Inc., La Jolla, CA, USA).

Continuous quantitative variables are presented as Mean ± Standard Deviation (SD), while discrete variables are expressed as the median and interquartile range (IQR). Qualitative variables are represented by the number of observations and the percentage distribution. The parametric distribution of numerical variables was assessed using the Shapiro–Wilk normality test. Group differences for continuous parametric variables were evaluated using Student’s *t*-test, while the Wilcoxon–Mann–Whitney U test was employed when appropriate. To mitigate the risk of type 1 error in multiple repeated measures, Bonferroni–Dunn correction was applied. Categorical variables were compared using Pearson’s chi-squared test. Statistical significance was defined as a *p*-value < 0.05.

## 3. Results

A total of 50 patients were included in the study and equally allocated between groups (Figure 2).

No clinically relevant differences were noticed between group characteristics (Table 1).

**Figure 3:**
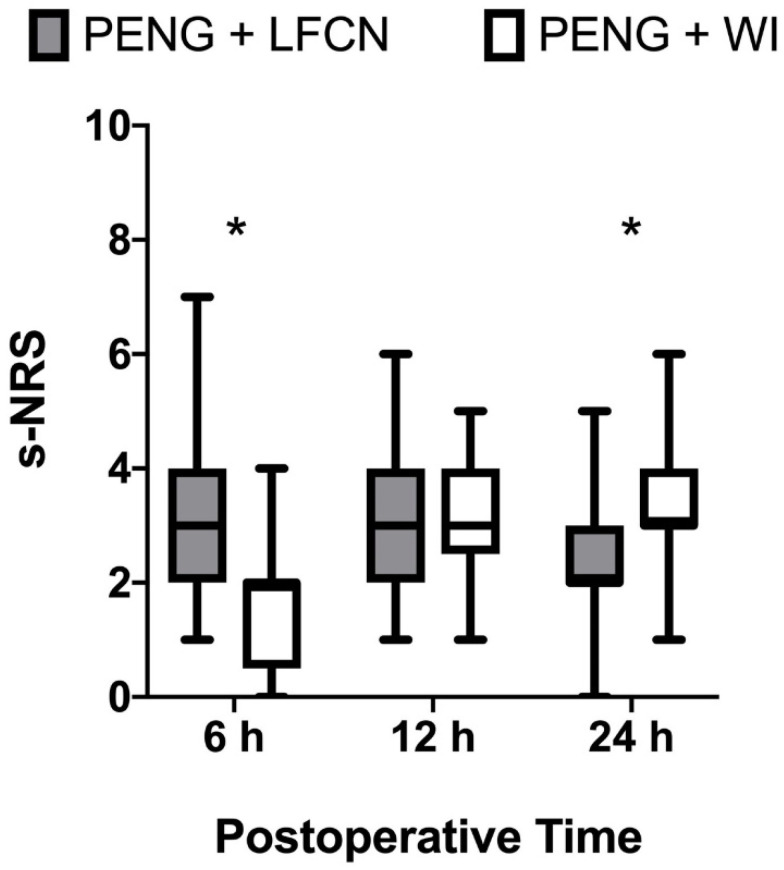
Static postoperative pain. The box plot shows postoperative pain scores in both study groups. Data include static pain reported at three different postoperative time points (6, 12 and 24 h). Pain severity is expressed using a 0–10 numeric rating scale, with 0 equal to no pain and 10 being the worst imaginable pain. Values are expressed as median (horizontal bars) with 25th–75th (box) and range of minimum to maximum value (whiskers); * denotes statistical significance (*p* < 0.05). PENG: pericapsular nerve group block; WI: wound infiltration; LFCN: lateral femoral cutaneous nerve block; s-NRS: numeric rating scale at rest (static).

The postoperative static NRS of patients receiving LFCN was higher than that of patients receiving WI at 6 h but lower at 24 h, with a median (IQR) of 3 (2–4) vs. 2 (1–2) (*p*< 0.001) and 2 (2–3) vs. 3 (3–4) (*p* = 0.02), respectively (Figure 3).

**Figure 4:**
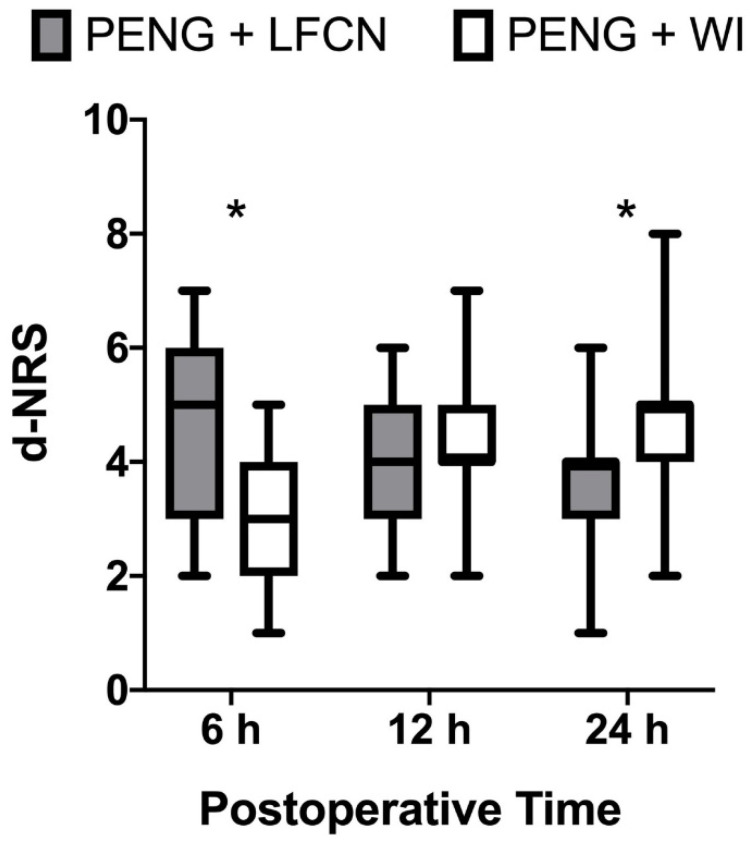
Dynamic postoperative pain. The box plot shows postoperative pain scores in both study groups. Data include dynamic pain reported at three different postoperative time points (6, 12 and 24 h). Pain severity is expressed using a 0–10 numeric rating scale, with 0 equal to no pain and 10 being the worst imaginable pain. Values are expressed as median (horizontal bars) with 25th–75th (box) and range of minimum to maximum value (whiskers); * denotes statistical significance (*p* < 0.05). PENG: pericapsular nerve group block; WI: wound infiltration. LFCN: lateral femoral cutaneous nerve block; d-NRS: numeric rating scale on movement (dynamic).

Static pain scores at 12 h did not show significant differences, with an NRS of 3 (2–4) for WI vs. 3 (3–4) for LFCN (*p* = 0.94). Dynamic pain scores followed a similar trend, showing a median NRS of 5 (3–6) vs. 3 (2–4) (*p* < 0.001), 4 (3–5) vs. 4 (4–5) (*p* = 0.18) and 4 (3–4) vs. 5 (4–5) (*p* = 0.019) at 6, 12, and 24 h, respectively (Figure 4).

Secondary outcomes are summarized in Table 2.

Total morphine consumption did not differ among groups, with a mean ± SD of 6.9 ± 3.6 mg for LCFN vs. 6.5 ± 4.6 for WI (*p* = 0.45). Regarding functional outcomes, the LFCN group showed a worse ROM at 6 h postoperatively (59.8° ± 11.2 vs. 71.4° ± 15.5, *p* = 0.011), while a better ROM was noticed at 24 h (71.4° ± 15.5 vs. 60.6 ± 16.5, *p* = 0.018), with no significant differences at 12 h (69.6° ± 9.3 vs. 62.8 ± 12.8, *p* = 0.1).

However, the ability to perform physiotherapy and ambulation during the first POD was comparable between groups, as well as the total length of stay (Table 2).

Moreover, no postoperative quadriceps paralysis was noticed except for one patient in the LFCN group and two patients in the WI group, but in all cases, it occurred only at 6 h postoperatively. Last, the incidence of postoperative complications was comparable and not significant.

## 4. Discussion

PENG block, despite being shown to be effective for postoperative analgesia following THA in different randomized clinical trials [14,19,20,21], is analgesically incomplete, as it does not cover the skin, which has the highest concentration of nociceptors involved in this kind of surgery. For this reason, several trials have investigated combining PENG block with other regional techniques, including periarticular infiltration, intra-articular injection and quadratus lumborum block [22,23,24,25,26].

In our study, both LFCN and WI ensured successful analgesia combined with PENG block, as no severe pain scores were noticed at any time point among the groups, although there were some differences. The WI group showed better analgesia and range of movements at 6 h after surgery compared to the LFCN group, which showed better scores at 24 h postoperatively. However, these differences did not impact opioid consumption or the remaining functional outcomes, including ambulation and physiotherapy. These results lead us to speculate about some clinical considerations, as they may reflect the strength and limitations of both techniques.

LCFN innervates only part of the skin involved in post-surgical pain following THA via the posterior approach, and this could justify the better analgesia in the WI group during the first postoperative hours. In contrast, better pain scores at 24 h postoperatively may be explained by the local anesthetic pharmacokinetics, which are characterized by a slower systemic absorption in the case of perineural rather than subcutaneous administration [27]. Another aspect to discuss is the discrepancy of ROM, which increases over time in the LFCN group, while it decreases in the WI group. As the articular ROM, in the absence of muscular block, may be strictly dependent on analgesia (in an indirectly proportional relationship with pain scores), it is reasonable to think that the inverted trend regarding ROM among the two groups reflects the same tendency of pain scores.

This is the first clinical trial analyzing the effect of PENG block compared to LFCN block or WI for postoperative analgesia after THA.

Gurbuz et al. explored a similar association through a prospective evaluation of 22 patients undergoing total hip replacement. They found a longer time for first analgesic demand in the WI group, although the LFCN group had lower pain scores at 24 h, similar to our study. However this study had important limitations including a lack of randomization, a poor sample size, and the surgical approach was not specified [28]. Dr. Liang and colleagues have recently demonstrated the superiority of PENG block combined with LFCN block vs. supra-inguinal FIB in improving postoperative analgesia after THA [8]. However, the study population was heterogeneous, as both fractured and non-fractured patients were included. Moreover, Dr. Liang compared the association of PENG plus LFCN block vs. SIFI block, which gives an indirect LFCN block itself, and for this reason, it seems unclear if any differences in outcomes can be attributed to the effect of LFCN block rather than PENG block.

Future studies may focus on the combination of PENG block with other techniques to enhance postoperative skin analgesia. In particular, the iliohypogastric nerve and subcostal nerve have been shown to innervate most of the skin involved in surgery, and they could easily be blocked through fascia transversalis or a lateral quadratus lumborum block [5].

At the same time, LFCN block could be added to WI to maximize the analgesic effect of both technique during the first 24 h, as suggested by this study, while being careful about the maximum recommended local anesthetic dosage.

In this study, we used 0.5% ropivacaine in our daily practice. We prefer this concentration to lower ones aiming to enhance block duration, as the rate of LA absorption (which impacts on block’s duration) also depends on the administered dosage [27], although no studies have yet analyzed the impact of different ropivacaine dosages on PENG block, LFCN block and WI in hip surgery. Despite a high concentration of ropivacaine, the total dosage administered to every patient (200 mg) was not superior to the maximum dosage recommended for one shot nerve block in adults (250 mg). Moreover, after administration of LA, patients were always monitored to exclude early prodromal symptoms related to Local Anesthetic Systemic Toxicity (LAST), like perioral paresthesia, metallic taste and tinnitus. In addition, clinical and ECG monitoring were maintained until discharge from PACU.

Regarding the duration of the WI analgesic effect, we think it may be prolonged through the use of a continuous technique or liposomal local anesthetics, as already demonstrated in different surgeries [13,29,30].

Last, we would like to discuss three cases (one in the LFCN group and two in the WI group) of postoperative quadriceps paralysis recorded at 6 h.

As described by a recent cadaveric study, this event could be related to the spread of local anesthetic to the femoral nerve during the execution of PENG block, especially when more than 13 mL is injected [31]. However, this has never been demonstrated to cause a significant impact on postoperative functional outcomes, including physiotherapy and ambulation. Not by chance, although Aliste et al. showed a similar incidence of quadriceps paralysis [19], it did not influence functional outcomes, and the same was true for our study. It is worth investigating the use of newer high definition US technology to better identify the optimal injection point and LA spread during the PENG block execution in order to further reduce the risk of femoral nerve involvement.

### Limitations

This study has several limitations.

Our actual institutional protocols require the patients to start ambulation after 24 h, and for this reason, it was not possible to investigate the ability of patients to walk before this time point. The same issue regards the ability to start of physiotherapy, which was assessed on POD 1. Future studies assessing these outcomes in earlier time points may overcome these limits.

Secondary outcomes including opioid consumption and length of stay did not show significant differences, although our sample size was powered 90% only for main outcomes: that means higher samples may be required to significantly evaluate the true effect of these outcomes. Moreover, we missed the assessment of other functional outcomes, i.e., the timed up and go (TUG) and walking tests, although its accuracy could be limited by different baseline health statuses and muscular tropisms among patients [32].

Pain scores were recorded only during the first 24 h after surgery, although, based on our clinical experience and our previous study, we consider this time interval to be the most critical for postoperative analgesia following THA via the posterior approach. Furthermore, we did not take the social differences among patients into account, and this could represent a potential bias in pain reporting.

Regarding the assessment of quadriceps strength, we did not use any muscular strength grading to differentiate between paralysis and paresis, as in other studies [19]. However, this method has been shown to have several limitations due to the variability in examiners’ subjective perceptions [33].

Our study focused on postoperative acute pain, although it would be interesting for future investigations to analyze the impact of these regional anesthesia techniques on chronic postoperative pain, whose incidence is estimated to be 10% for THA [34]. In this study, we used multimodal analgesia through NSAIDs, which positively impacts postoperative pain and may overestimate the efficacy of a regional anesthesia technique. However, NSAIDs “around the clock” has been applied to both groups at the same dosages, and this is reasonably unlikely to represent a bias.

Lastly, although we monitored clinical and vital signs to exclude LAST, we did not measure systemic absorption rates. Future studies should look at blood levels of ropivacaine after PENG block in order to confirm the safety of this technique and investigate other possible mechanism of actions (systemic?).

## 5. Conclusions

LFCN block was not inferior to WI for postoperative analgesia and functional recovery in association with PENG block during the first postoperative day, although it had worse short-term pain scores.

However, no significant differences were observed in functional recovery and opioid consumption. Based on these results, it is possible to consider both LFCN block and WI as valuable options to cause skin analgesia after THA. Moreover, we hypothesize that an association of LFCN block and WI combined with PENG block may enhance the analgesic effect of both of the techniques during the first 24 h. However, future studies are expected to confirm this hypothesis and find the best combination between PENG block and other techniques to enhance analgesia and functional outcomes after THA.

## Figures and Tables

**Table 1:** Patient characteristics.

	PENG + LFCN (n = 25)	PENG + WI (n = 25)
Age (yrs)	67.7 ± 8.8	65.2 ± 13
Sex (M/F)	12/13	14/11
BMI (kg/m^2^)	27.9 ± 3.7	28.5 ± 5.3
ASA score, n (%) - I - II - III	1 (4%) 13 (52%) 11 (44%)	2 (8%) 16 (64%) 8 (32%)
Chronic Opiate Use, n (%) - Yes - No	3 (12%) 22 (88%)	4 (16%) 21 (84%)
Surgery Duration (min)	107 ± 20	105 ± 18

Values are reported as number (percentage) of subjects and mean ± standard deviation (SD). PENG: pericapsular nerve group block; LFCN: lateral femoral cutaneous nerve block; WI: wound infiltration; BMI: body mass index.

**Table 2:** Secondary outcomes.

	PENG + LFCN (n = 25)	PENG + WI (n = 25)	*p* Value
Morphine Consumption (mg)			
- 24 h - Total	4.3 ± 3.1 6.9 ± 3.6	3.7 ± 2.9 6.5 ± 4.6	0.57 0.45
Range of Movement (°)			
- 6 h - 12 h - 24 h	59.8 ± 11.2 69.6 ± 9.3 71.6 ± 9.9	71.4 ± 15.5 62.8 ± 12.8 60.6 ± 16.5	0.011 0.1 0.018
Quadriceps Paralysis 6/12/24 h, n (%)	1 (4%)/0/0	2 (8%)/0/0	>0.9
Ability to perform physiotherapy at POD1, n (%)	25 (100%)	25 (100%)	>0.9
Ability to ambulation at POD1, n (%)	25 (100%)	24 (96%)	>0.9
Length of stay (days)	2 (2–3)	2 (2–4)	0.96
Nausea/vomiting, n (%)	1 (4%)	2 (8%)	>0.9
Dizziness, n (%)	0	0	-
Vascular Puncture, n (%)	0	0	-

Values are reported as number (percentage) of subjects, mean ± standard deviation (SD) or median and interquartile range (IQR). LFCN: lateral femoral cutaneous nerve block; POD: postoperative day; WI: wound infiltration; PENG: pericapsular nerve group block.

## Data Availability

Data are available upon reasonable request. Please contact the corresponding author (Alessandro Strumia: a.strumia@policlinicocampus.it).
